# LocationSpark: In-memory Distributed Spatial Query Processing and Optimization

**DOI:** 10.3389/fdata.2020.00030

**Published:** 2020-10-16

**Authors:** Mingjie Tang, Yongyang Yu, Ahmed R. Mahmood, Qutaibah M. Malluhi, Mourad Ouzzani, Walid G. Aref

**Affiliations:** ^1^Chinese Academy of Science, Beijing, China; ^2^Facebook, Menlo Park, CA, United States; ^3^Department of Computer Science, Purdue University, West Lafayette, IN, United States; ^4^KINDI Center for Computing Research, Qatar University, Doha, Qatar; ^5^Qatar Computing Research Institute, HBKU, Doha, Qatar

**Keywords:** spatial data, query processing, in-memory computation, parallel computing, query optimization

## Abstract

Due to the ubiquity of spatial data applications and the large amounts of spatial data that these applications generate and process, there is a pressing need for scalable spatial query processing. In this paper, we present new techniques for spatial query processing and optimization in an in-memory and distributed setup to address scalability. More specifically, we introduce new techniques for handling query skew that commonly happens in practice, and minimizes communication costs accordingly. We propose a distributed query scheduler that uses a new cost model to minimize the cost of spatial query processing. The scheduler generates query execution plans that minimize the effect of query skew. The query scheduler utilizes new spatial indexing techniques based on bitmap filters to forward queries to the appropriate local nodes. Each local computation node is responsible for optimizing and selecting its best local query execution plan based on the indexes and the nature of the spatial queries in that node. All the proposed spatial query processing and optimization techniques are prototyped inside Spark, a distributed memory-based computation system. Our prototype system is termed LocationSpark. The experimental study is based on real datasets and demonstrates that LocationSpark can enhance distributed spatial query processing by up to an order of magnitude over existing in-memory and distributed spatial systems.

## 1. Introduction

Spatial computing is becoming increasingly important with the proliferation of mobile devices. Besides, the growing scale and importance of location data have driven the development of numerous specialized spatial data processing systems, e.g., SpatialHadoop (Eldawy and Mokbel, 2015), Hadoop-GIS (Aji et al., 2013). By taking advantage of the power and cost-effectiveness of MapReduce, these systems typically outperform spatial extensions on top of relational database systems by orders of magnitude (Aji et al., 2013). MapReduce-based systems allow users to run spatial queries using predefined high-level spatial operators without worrying about fault tolerance or computation distribution. However, these systems have the following two main limitations: (1) They do not leverage the power of distributed memory, and (2) They are unable to reuse intermediate data (Zaharia, 2016). Nonetheless, data reuse is very common in the spatial data processing. Spatial datasets, e.g., Open Street Map (OSM, for short, >60 G) and Point of Interest

(POI, for short, >20 G) (Eldawy and Mokbel, 2015), are relatively large in size. It is unnecessary to read these datasets continuously from disk [e.g., using HDFS; Shvachko et al., 2010] to respond to user queries. Moreover, intermediate query results have to be written back to HDFS, thus directly impeding the performance of further data analysis steps.

One way to address the above challenges is to develop an efficient execution engine for large-scale spatial data computation based on a memory-based computation framework (in this case, Spark; Zaharia, 2016). Spark is a computation framework that allows users to work on distributed in-memory data without worrying about data distribution or fault-tolerance. Recently, various Spark-based systems have been proposed for spatial data analysis, e.g., GeoSpark (Yu et al., 2015), Simba (Xie et al., 2016), and LocationSpark (Tang et al., 2016).

**Figure 1:**
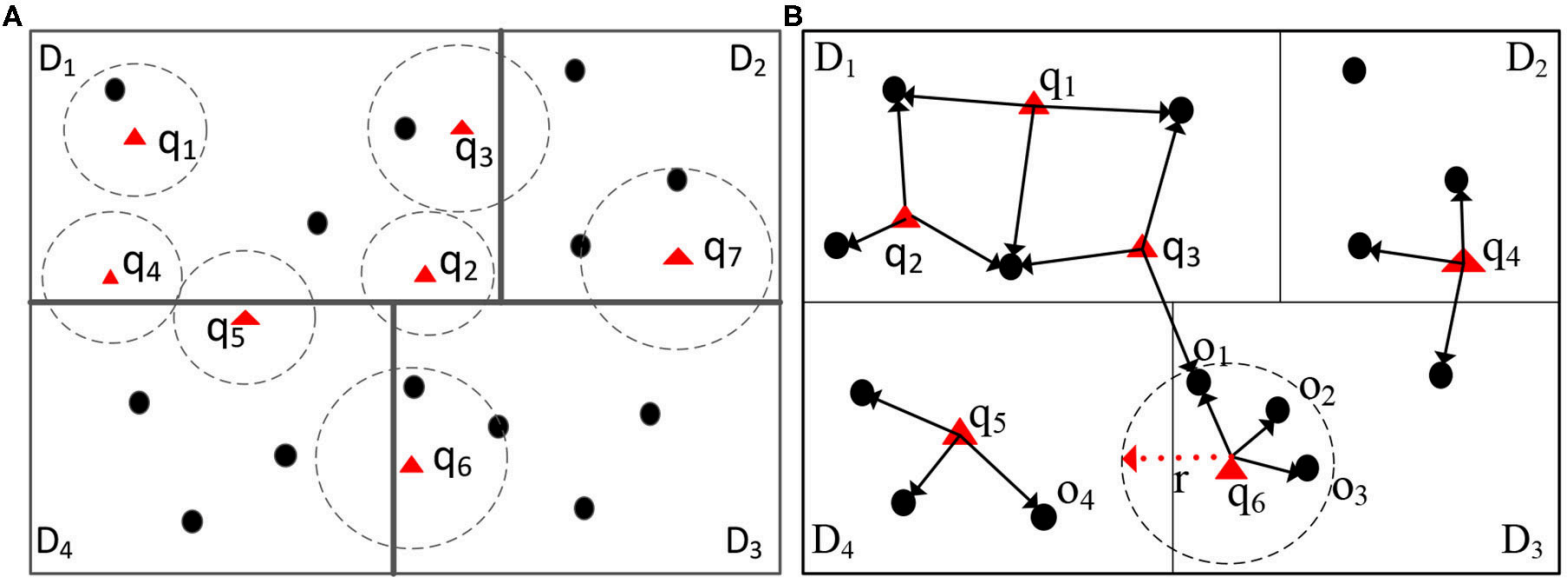
Illustration of spatial join and kNN join operators. Circles centered around the triangle focal points form one dataset, and the black dots form the second dataset. Spatial range join returns (dot, triangle) pairs when the dot is inside the circle. kNN join returns (triangle, dot) pairs when the dot is among the three nearest dots to the triangle. **(A)** Range join, **(B)** 3NN join.

Although addressing several challenges in spatial query processing, none of the existing systems can overcome the computation skew introduced by spatial queries. “Spatial query skew” is observed in distributed environments during spatial query processing when certain data partitions are overloaded by spatial queries. Traditionally, distributed spatial computing systems (e.g., Aji et al., 2013; Eldawy and Mokbel, 2015) first, learn the spatial data distribution by sampling the input data. Afterward, spatial data is partitioned evenly into equal-size partitions. For example, in Figure 1, the data points with dark dots are distributed evenly into four partitions. However, the query workload is what may cause computation skew. Given the partitioned data, consider two spatial join operators, namely range and *k*NN joins, that combine two datasets, say *D* and *Q*, with respect to a spatial relationship. For each point *q* ∈ *Q*, a spatial range join [Figure 1 (left)] returns data points in *D* that are inside the circle centered at *q*. In contrast, a *k*NN join [Figure 1 (right)] returns the *k* nearest-neighbors from the dataset *D* for each query point *q* ∈ *Q*. Both spatial operators are expensive, and may incur computation skew in certain workers, thus greatly degrading the overall performance.

For illustration, consider a large spatial dataset, with millions of points of interests (POIs), that is partitioned into different computation nodes based on the spatial distribution of the data, e.g., one data partition represents data from San Francisco, CA, and another represents data from Chicago, IL. Assume that we have incoming queries from people looking for different POIs, e.g., restaurants, train, or bus stations, and grocery stores, around their locations. These spatial range queries are consolidated into batches to be joined via an index on the POI data (e.g., using indexed nested-loops join). After partitioning the incoming spatial queries based on their locations, we observe the following issues: During rush hours in San Francisco from 4 p.m. to 6 p.m. (PST), San Francisco's data partition may encounter more queries than the data partition in Chicago, since it is already evening time in Chicago. Without an appropriate optimization technique, the data partition for San Francisco will take much longer time to process its corresponding queries while the workers responsible for the Chicago partition is lightly loaded. Similarly, if in Figure 1, the data points (the dark dots) correspond to the GPS records of Uber's cars, where multiple users (the triangles) are looking for the Uber carpool service around them. Partition *D*_1_, that corresponds, say to an airport, experiences more queries than the other partitions because people may prefer using Uber at this location. Being aware of the spatial query skew provides a new opportunity to optimize queries in distributed spatial data environments. The skewed partitions have to be assigned more computation power to reduce the overall processing time.

Furthermore, communication cost, generally a key factor of the overall performance, may become a bottleneck. When a spatial query touches more than one data partition, it may be the case that some of these partitions do not contribute to the final query result. For example, in Figure 1 (left part), queries *q*_2_, *q*_3_, *q*_4_, and *q*_5_ overlap more than one data partition (*D*_1_, *D*_2_, and *D*_4_). Traditionally, system would think these partitions would contribute the final query results, however, these partitions do not contain data points that satisfy the queries, since these queries overlap partitions only. Thus, scheduling queries (e.g., *q*_4_ and *q*_5_) to the overlapping data partition *D*_4_ incurs unnecessary communication costs. More importantly, for the spatial range join or *k*NN join operators over two large datasets, the cost of the network communication may become prohibitive without proper optimization.

In this paper, we introduce LocationSpark, an efficient memory-based distributed spatial query processing system. LocationSpark has a query scheduler to mitigate query skew. The query scheduler uses a cost model to analyze the skew for use by the spatial operators, and a plan generation algorithm to construct a load-balanced query execution plan. After plan generation, local computation nodes select the proper algorithms to improve their local performance based on the available spatial indexes and the registered queries on each node. Finally, to reduce communication cost when dispatching queries to their overlapping data partitions, LocationSpark adopts a new spatial bitmap filter, termed sFilter, that can speed up query processing by avoiding needless communication with data partitions that do not contribute to the query answer. We implement LocationSpark as a library in Spark that provides an API for spatial query processing and optimization based on Spark's standard dataflow operators.

The main contributions of this paper is as follows:

1. We present LocationSpark, a spatial computing system for the efficient processing of spatial queries in a distributed in-memory environment.
2. We address data and query skew issues to improve load balancing while executing spatial operators, e.g., spatial range joins and *k*NN joins. LocationSpark produces cost-optimized query execution plans over in-memory distributed spatial data.
3. We introduce a new lightweight yet efficient spatial bitmap filter to reduce communication costs.
4. We realize the introduced query processing and optimization techniques inside LocationSpark, and conduct a large-scale evaluation on real spatial data and common benchmark algorithms. We compare LocationSpark against state-of-the-art distributed spatial data processing systems. Experimental results illustrate an enhancement in performance by up to an order of magnitude over existing in-memory distributed spatial query processing systems.

The rest of this paper proceeds as follows. Section 2 presents the problem definition and an overview of distributed spatial query processing. Section 3 introduces the cost model and the cost-based query plan scheduler and optimizer and their corresponding algorithms. Section 4 presents an empirical study for local execution plans in local computation nodes. Section 5 introduces the spatial bitmap filter, and explains how it can speedup spatial query processing in a distributed setup. The experimental results are presented in section 6. Section 7 discusses the related work. Finally, section 8 concludes the paper.

## 2. Preliminaries

### 2.1. Data Model and Spatial Operators

LocationSpark stores spatial data as key-value pairs. A tuple, say *o*_*i*_, contains a spatial geometric key *k*_*i*_ and a related value *v*_*i*_. The spatial data type for key *k*_*i*_ can be a two-dimensional point, e.g., latitude-longitude, a line-segment, a poly-line, a rectangle, or a polygon. The value types *v*_*i*_ is specified by the user, e.g., a text data type if the data tuple is a tweet. In this paper, we assume that queries are issued continuously by users, and are processed by the system in batches (e.g., similar to the DStream model; Zaharia, 2016).

LocationSpark supports various types of spatial query predicates including spatial range search, *k*-NN search, spatial range join, and *k*NN join. In this paper, we focus our discussion on the spatial range join and *k*NN join operators on two datasets, say *Q* and *D*, the form the outer and inner tables, respectively.

*Definition 1.* **Spatial Range Select**—*range*(*q,D*): Given a spatial range area *q* (e.g., circle or rectangle) and a dataset *D*, *range*(*q,D*) finds all tuples from *D* that overlap the spatial range de fined by *q*.

*Definition 2.* **Spatial Range Join**—*Q⋈*_sj_*D*: Given two datasets *Q* and *D*, *Q⋈*_sj_*D*, combines each object *q* ∈ *Q* with its range search results from *D*, *Q⋈*_sj_*D*= {(*q, o*)|*q* ∈ *Q, o* ∈ *range*(*q,D*)}.

*Definition 3.* **kNN Select**—*k*NN(q,D): Given a query tuple *q*, a dataset *D*, and an integer *k*, *k*NN(q,D), returns the output set {*o*|*o* ∈ *D* and ∀*s* ∈ *D* and *s*≠*o*, ||*o, q*|| ≤ ||*s, q*||}, where the number of output objects from *D*, |*kNN*(*q,D*)| = *k*.

*Definition 4.* **kNN Join**—*Q⋈*_knn_*D*: Given a parameter *k*, *k*NN join of *Q* and *D* computes for each object *q* ∈ *Q* its *k*-nearest-neighbors in *D*. *Q⋈*_knn_*D*= {(*q, o*)|∀*q* ∈ *Q*, ∀*o* ∈ *kNN*(*q,D*)}.

### 2.2. Overview of In-memory Distributed Spatial Query Processing in LocationSpark

**Figure 2:**
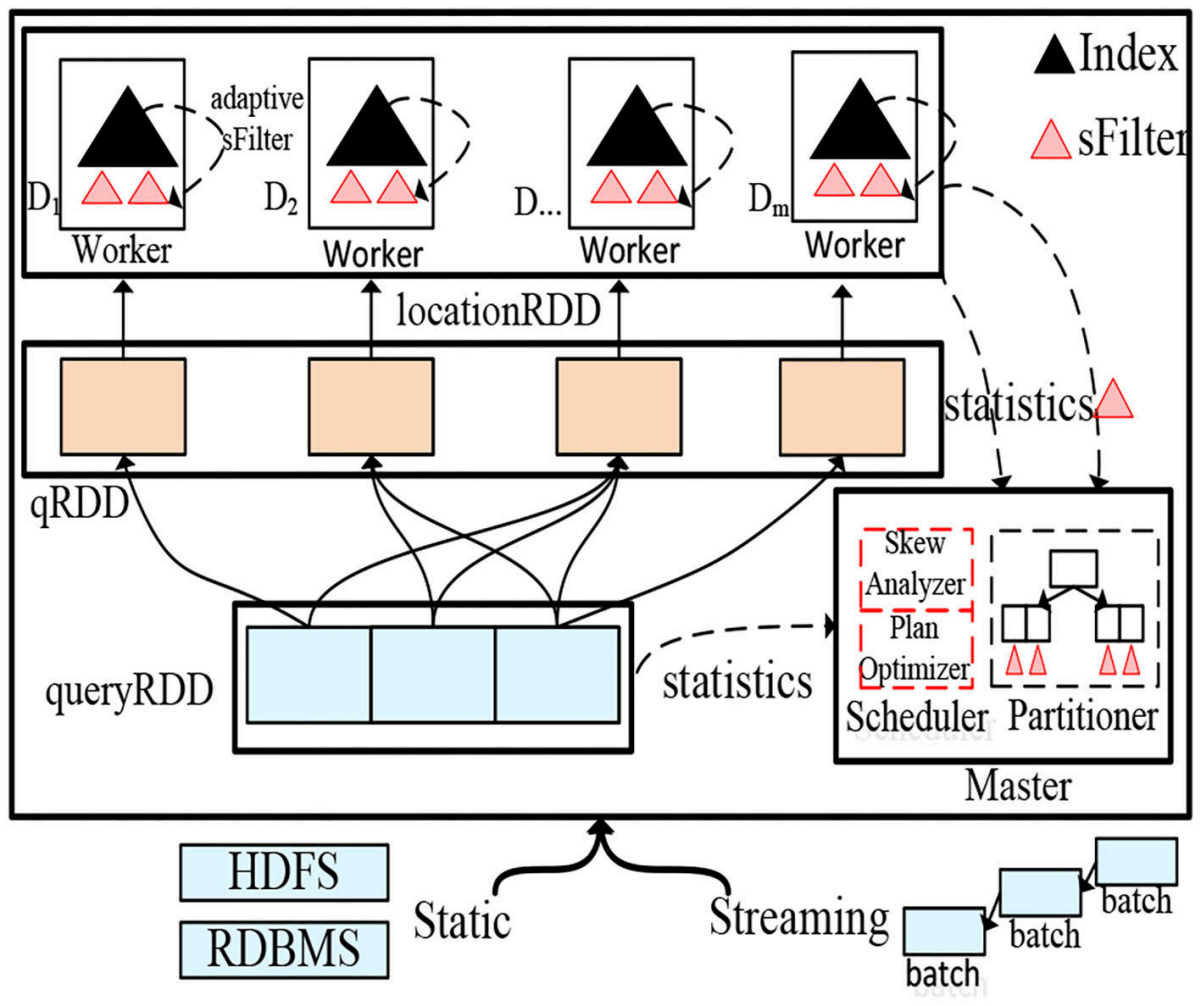
LocationSpark System architecture.

To facilitate spatial query processing, in LocationSpark, we construct a distributed spatial index for in-memory spatial data. Given a spatial dataset *D*, we obtain samples from *D*, and construct over these samples a spatial index (e.g., an R-tree) with *N* leaves. We refer to this index on the sample data as the *global spatial index*. It partitions *D* via data shuffling into *N* partitions. The global spatial index guarantees that each data partition approximately has the same amount of data. Then, each worker *W*_*i*_ of the *N* workers has a local data partition *D*_*i*_ that is roughly 1/*N*th of the data and constructs a local spatial index. Finally, the indexed data (termed the LocationRDD) is cached into memory. Figure 2 gives the architecture of LocationSpark and the physical representation of the partitioned spatial data based on the procedure outlined above, where the master node (e.g., Master in the Figure) stores the global spatial index that indexes the data partitions, while each worker has a local spatial index (e.g., the black triangle in the Figure) over the local spatial data within the partition. Notice that the global spatial index partitions the data into LocationRDDs as in Figure 2, and this index can be copied into various workers to help partition the data in parallel. The type of each local index, e.g., a Grid, an R-tree, or an IR-tree, for a data partition can be determined based on the specifics of the application scenarios. For example, a Grid can be used for indexing moving objects while an R-tree can be used for polygonal objects. For spatial range join, two strategies are possible; either replicate the outer table and send it to the node where the inner table is, or replicate the inner table data and send it to the different processing nodes where the outer table tuples are. In a shared execution, the outer table is typically a collection of range query tuples, and the inner table is the queried dataset. If this is the case, it would make more sense to send the outer table of queries to the inner data tables as the outer table is usually much smaller compared to the inner data tables. In this paper, we adopt this technique because it would be impracticable to replicate and forward copies of the large inner data table.

Thus, each tuple *q* ∈ *Q* is replicated and forwarded to the partitions that spatially overlap with it. These overlapping partitions are identified using the global index. Then, a post-processing step merges the local results to produce the final output. For example, we replicate *q*_2_ in Figure 1 (left part) and forward it to data partitions *D*_1_, *D*_3_, and *D*_4_. Then, we execute a spatial range search on each data partition locally. Next, we merge the local results to form the overall output of tuple *q*2. As illustrated in Figure 2, the outer table that corresponds to a shared execution plan's collection of queries (termed queryRDD) are first partitioned into qRDD based on the overlap between the queries in qRDD and the corresponding data partitions. Then, the local search takes place over the local data partitions of LocationRDD.

The *k*NN join operator is implemented similarly in a simple two-round process. First, each outer focal point *q*_*i*_ ∈ *Q* is transferred to the worker that holds the data partition that *q*_*i*_ spatially belongs to. Then, the *k*NN join is executed locally in each data partition, producing the *k*NN candidates for each focal point *q*_*i*_. Afterward, the maximum distance from *q*_*i*_ to its *k*NN candidates, say radius *r*_*i*_, is computed. If the radius *r*_*i*_ overlaps multiple data partitions, Point *q*_*i*_ is replicated into these overlapping partitions, and another set of *k*NN candidates is computed in each of these partitions. Finally, we merge the *k*NN candidates from the various partitions to get the exact result. For example, in Figure 1 (right), assume that we want to evaluate a 3NN query for Point *q*_6_. The first step is to find the 3NN candidates for *q*_6_ in data Partition *D*_3_. Next, we find that the radius *r* for the 3NN candidates from Partition *D*_3_ overlaps Partition *D*_4_. Thus, we need to compute the 3NN of *q*_6_ in Partition *D*_4_ as well. Notice that the radius *r* can enhance the 3NN search in Partition *D*_4_ because only the data points within Radius *r* are among the 3NN of *q*_6_. Finally, the 3NN of *q*_6_ are *o*_1_, *o*_2_, and *o*_3_.

### 2.3. Challenges

The outer and inner tables (or, in shared execution terminology, the queries, and the data) are spatially collocated in distributed spatial query processing. We refer to the outer table as being the queries table, e.g., containing the ranges of range operations, or the focal points of *k*NN operations. Assume further that the outer (queries) table is the smaller of the two. We refer to the inner table by the data table (in the case of shared execution of multiple queries together). The distribution of the incoming spatial queries (in the outer tables) changes dynamically over time, with bursts in certain spatial regions. Thus, evenly distributing the input data *D* to the various workers may result in a load imbalance at times. LocationSpark's scheduler identifies the skewed data partitions based on a cost model, repartitions, and redistributes the data accordingly, and selects the optimal repartitioning strategies for both the outer and inner tables, and consequently generates an overall optimized execution plan.

Communication cost is a major factor that affects system performance. LocationSpark adopts a spatial bitmap filter to reduce network communication costs. The role of the spatial bitmap filter is to prune the data partitions that overlap the spatial ranges from the outer tables but do not contribute to the final operation's results. This spatial bitmap filter is memory-based and is space- and time-efficient. The spatial bitmap filter adapts its structure as the data and query distributions change.

## 3. Query Plan Scheduler

This section addresses how to dynamically handle query skew (the outer table of the join). First, we present the cost functions for query processing and analyze the bottlenecks. Then, we show how to repartition the skewed data partitions to speed up processing. This is formulated as an optimization problem that we show is NP-complete. Consequently, we introduce an approximation algorithm to solve the data skew repartitioning problem. Although presented for spatial range joins, the proposed technique all applies to *k*NN joins.

### 3.1. The Cost Model

The inner table *D* of the spatial range join is distributed into *N* data partitions, where each partition *D*_*i*_ is indexed and is cached in memory. For the query table *Q* (i.e., the outer table of spatial range join), each query *q*_*i*_ ∈ *Q* is shuffled to the data partitions that spatially overlap with it. Let ϵ(*Q, N*) be the shuffling cost, and *E*(*D*_*i*_) be the execution time of local queries at Partition *D*_*i*_. The execution times of local queries depend on the queries and the built indexes. The estimation of *E*(*D*_*i*_) is presented later. After the local results are computed, the post-processing step merges the local results to produce the final output. The corresponding cost is denoted by ρ(*Q*).

Overall, the runtime cost for the spatial range join operation is:

(1) $$\begin{array}{r} C\left(D,Q\right)=\epsilon \left(Q,N\right)+\underset{i\in \left[1,N\right]}{\max}E\left(D_{i}\right)+\rho \left(Q\right), \end{array}$$

where *N* is the number of data partitions. In reality, the cost of query shuffling is far less than the other costs as the number of queries is much smaller than the number of data items. Thus, the runtime cost can be estimated as follows:

(2) $$\begin{array}{r} C\left(D,Q\right)=\underset{i\in \left[1,N\right]}{\max}E\left(D_{i}\right)+\rho \left(Q\right) \end{array}$$

We categorize the data partitions into two types: skewed (*D*^*s*^) and non-skewed (*D*^*ns*^). The execution time of the local queries in the skewed partitions is the bottleneck. The runtime costs for skewed and non-skewed data partitions are $\underset{i\in \left[1,\hat{N}\right]}{\max}E\left(D_{i}^{s}\right)$ and $\underset{j\in \left[1,\bar{N}\right]}{\max}E\left(D_{j}^{ns}\right)$, respectively, where $\hat{N}$ (and $\bar{N}$) is the number of skewed (and non-skewed) data partitions, and $N=\hat{N}+\bar{N}$. Thus, Equation (2) can be rewritten as follows:

(3) $$\begin{array}{r} C\left(D,Q\right)=\max\left\{\underset{i\in \left[1,\hat{N}\right]}{\max}E\left(\overset{s}{\underset{i}{D}}\right),\underset{j\in \left[1,\bar{N}\right]}{\max}E\left(\overset{ns}{\underset{j}{D}}\right)\right\}+\rho \left(Q\right) \end{array}$$

### 3.2. Execution Plan Generation

**Figure 3:**
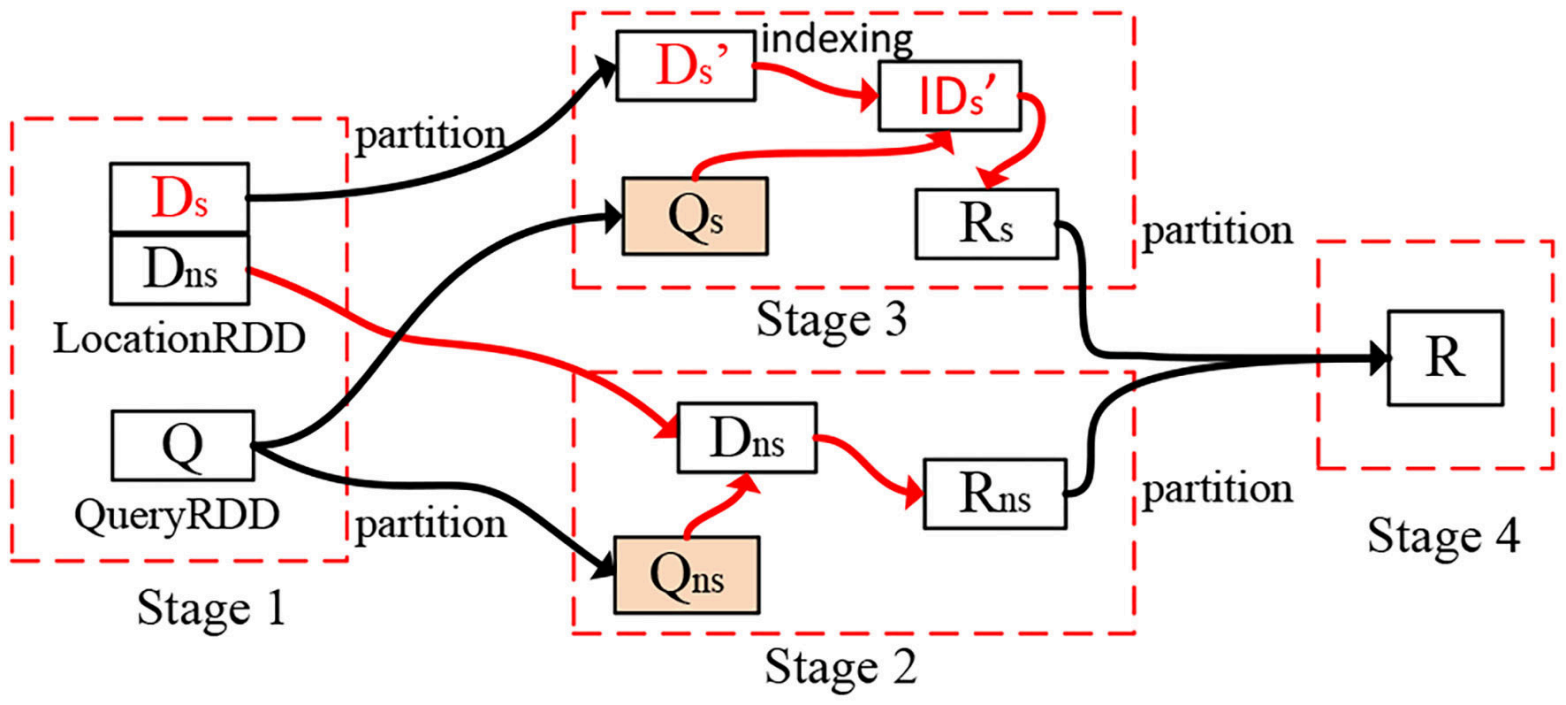
Execution plan for spatial range join. The red lines identify local operations, and black lines show the data partitioning.

The goal of the query scheduler is to minimize the query processing time subject to the following constraints: (1) The limited number of available resources (i.e., the number of partitions) in a cluster, and (2) The overhead of network bandwidth and disk I/O. Given the partitioned and indexed spatial data, the cost estimators for query processing based on sampling (that is introduced below), and the available number of data partitions, the optimizer returns an execution plan that minimizes query processing time. First, the optimizer determines if any partitions are skewed. Then, it repartitions them subject to the introduced cluster and networking constraints. Finally, the optimizer evaluates the plan on the newly repartitioned data to determine whether or not it minimizes query execution time (see Figure 3). For example, the partition D is repartitioned into two parts for skew issues.

Estimating the runtime cost of executing the local queries and the cost of merging the final results is not straightforward. The local query processing time *E*(*D*_*i*_) is influenced by various factors including the types of spatial indexes used, the number of data points in *D*_*i*_, the number of queries directed to *D*_*i*_, related spatial regions, and the available memory. Similar to Kwon et al. (2010), we assume that the related cost functions are monotonic, and can be approximated using samples from the outer and inner tables (queries and data). Thus, the local query execution time is formulated as follows: $E\left(D_{i}\right)=E_{s}\left(\tilde{D_{i}},\tilde{Q_{i}},\alpha ,A\right)$, where $\tilde{D_{i}}$ is a sample of the original inner table dataset, $\tilde{Q_{i}}$ is the sample of the outer table queries, *A* is the area of the underlying spatial region, and α is the sample ratio to scale up the estimate to the entire dataset. After computing a representative sample of the data points and queries, e.g., using reservoir sampling (Vitter, 1985), the cost function *E*(*D*_*i*_) estimates the query processing runtime in Data Partition *D*_*i*_. More details on computing *E*(*D*_*i*_), ρ(*Q*_*i*_), and the sample size can be found in Kwon et al. (2010).

Given the estimated runtime cost over skewed and non-skewed partitions, the optimizer splits one skewed data Partition $D_{i}^{s}$ into *m*′ data sub-partitions. Assume that $\hat{Q_{i}}$ is the set of queries originally assigned to Partition $D_{i}^{s}$. Let the overheads due to data shuffling, re-indexing, and merging be $\beta \left(D_{i}^{s}\right)$, $\gamma \left(D_{i}^{s}\right)$, and $\rho \left(\hat{Q_{i}}\right)$, respectively. After splitting a skewed partition ($D_{i}^{s}$), the new runtime is:

(4) $$\begin{array}{r} \hat{E\left(D_{i}^{s}\right)}=\beta \left(D_{i}^{s}\right)+\underset{s\in \left[1,m^{\prime }\right]}{\max}\left\{\gamma \left(D_{s}\right)+E\left(D_{s}\right)\right\}+\rho \left(\hat{Q_{i}}\right). \end{array}$$

Hence, we can split $D_{i}^{s}$ into multiple partitions only if $\hat{E\left(D_{i}^{s}\right)}$ $<E\left(D_{i}^{s}\right)$. As a result, the new query execution time $\hat{C\left(D,Q\right)}$ is:

(5) $$\begin{array}{r} \hat{C\left(D,Q\right)}=\max\left\{\underset{i\in \left[1,\hat{N}\right]}{\max}\left\{\hat{E\left(\overset{s}{\underset{i}{D}}\right)}\right\},\underset{j\in \left[1,\bar{N}\right]}{\max}\left\{E\left(\overset{ns}{\underset{j}{D}}\right)\right\}\right\}+\rho \left(\bar{Q}\right). \end{array}$$

Thus, we can formulate the query plan generation based on the data repartitioning problem as follows:

*Definition 5*. Let *D* be the set of spatially indexed data partitions, *Q* be the set of spatial queries, *M* be the total number of data partitions, and their corresponding cost estimation functions, i.e., local query execution *E*(*D*_*i*_), data repartitioning β(*D*_*i*_), and data indexing cost estimates γ(*Q*_*i*_). The query optimization problem is to choose a skewed Partition *D*^*s*^ from *D*, repartition each $D_{i}^{s}\in D^{s}$ into multiple partitions, and assign spatial queries to the new data partitions. The new data partition set, say *D*′, contains partitions $D_{1}^{\prime },D_{2}^{\prime },\ldots ,D_{k}^{\prime }$. s.t. (1) $\hat{C\left(D,Q\right)}<C\left(D,Q\right)$ and (2) |*D*′| ≤ *M*.

Unfortunately, as we show below, this problem is NP-complete. In the next section, we present a greedy algorithm for this problem.

**Theorem 1**. *Optimized query plan generation with data repartitioning for distributed indexed spatial data is NP-complete*.

The proof is given in Mingjie et al. (2016).

### 3.3. A Greedy Algorithm

The general idea is to search for skewed partitions based on their local query execution times. Then, we split the selected data partitions only if the runtime can be improved. If the runtime cannot be improved, or if all the available data partitions are consumed, then the algorithm terminates. While this greedy algorithm cannot guarantee optimal query performance, our experiments show significant improvement (by one order of magnitude) over the plan executing on the original partitions. Algorithm 1 gives the pseudocode for the greedy partitioning procedure.


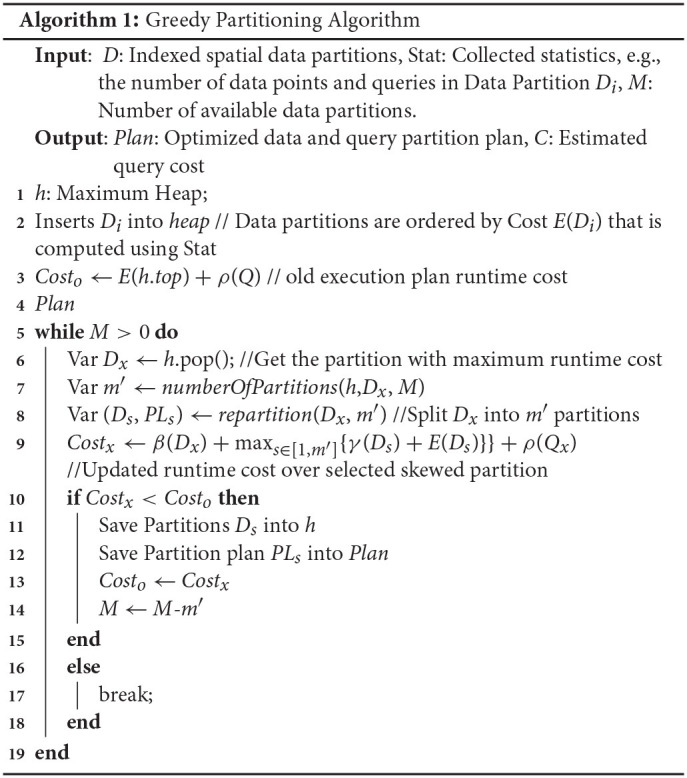


Algorithm 1 includes two functions, namely *numberOfPartitions* and *repartition*. Function *numberOfPartitions* in line 7 computes the number of sub-partitions *m*′ that the skewed partition will be split into. We could split a skewed partition into two partitions each time, but this is not necessarily efficient. Given data partitions *D* = {*D*_1_, *D*_2_, …, *D*_*N*_}, let Partition *D*_1_ be the one with the largest local query execution time *E*(*D*_1_). From Equation (2), the execution time is approximated by *E*(*D*_1_)+ρ(*Q*). To reduce this execution time, *D*_1_ is split into *m*′ partitions, and the query execution time for *D*_1_ is updated to $\hat{E\left(D_{1}\right)}$. For all other partitions *D*_*i*_ ∈ *D* (*i*≠1), the runtime is the $\max\left\{E\left(D_{i}\right)\right\}+\rho \left(Q^{\prime }\right)=\Delta$, where *i* = [2, …, *N*] and *Q*′ are the queries related to all data partitions except *D*_1_. Thus, the runtime is $\max\left\{\Delta ,\hat{E\left(D_{1}\right)}\right\}$, and is improved if

(6) $$\begin{array}{r} \max\left\{\Delta ,\hat{E\left(D_{1}\right)}\right\}<E\left(D_{1}\right)+\rho \left(Q\right) \end{array}$$

As a result, we need to compute the minimum value of *m*′ to satisfy Equation (6), since Δ, *E*(*D*_1_), and ρ(*Q*) are known.

Function *repartition* in line 8 splits the skewed data partitions and reassigns the spatial queries to the new data partitions using two strategies. The first strategy repartitions based on data distribution. Because each data partition *D*_*i*_ is already indexed by a spatial index, the data distribution can be learned directly by recording the number of data points in each branch of the index. Then, we repartition data points in *D*_*i*_ into multiple parts based on the recorded numbers while guaranteeing that each newly generated sub-partition contains an equal amount of data. In the second strategy, we repartition a skewed Partition *D*_*i*_ based on the distribution of the spatial queries. First, we collect a sample *Q*_*s*_ from the queries *Q*_*i*_ that are originally assigned to Partition *D*_*i*_. Then, we compute how *Q*_*s*_ is distributed in Partition *D*_*i*_ by recording the frequencies of the queries as they belong to branches of the index over the data. Thus, we can repartition the indexed data based on the query frequencies. Although the data sizes may not be equal, the execution workload will be balanced. In our experiments, we choose this second approach to overcome query skew. To illustrate how the proposed query-plan optimization algorithm works, consider the following example.

**Running Example**. Given data partitions *D* = {*D*_1_, *D*_2_, *D*_3_, *D*_4_, *D*_5_}, where the number of data points in each partition is 50, the number of queries in each partition *D*_*i*_, 1 ≤ *i* ≤ 5 is 30, 20, 10, 10, and 10, respectively, and the available data partitions *M* is 5. For simplicity, the local query processing cost is *E*(*D*_*i*_) = |*D*_*i*_| × |*Q*_*i*_| × *p*_*e*_, where *p*_*e*_ = 0.2 is a constant. The cost of merging the results is ρ(*Q*) = |*Q*| × λ × *p*_*m*_, where *p*_*m*_ = 0.05, and λ = 10 is the approximate number of retrieved data points per query. The cost of data shuffling and re-indexing after repartitioning is $\beta \left(D_{i},m^{\prime }\right)=|D_{i}|\times m^{\prime }\times p_{r}$, and γ(*D*_*s*_) = |*D*_*s*_| × *p*_*x*_, respectively, where *p*_*r*_ = 0.01 and *p*_*x*_ = 0.02. Without any optimization, from Equation (2), the estimated runtime cost for this input table is 340. LocationSpark optimizes the workload as follows. At first, it chooses Data Partition *D*_1_ as the skewed partition to be repartitioned because *D*_1_ has the highest local runtime (300), while the second largest cost is *D*_2_'s (200). Using Equation (6), we split *D*_1_ into two partitions, i.e., *m*′ = 2. Thus, Function repartition splits *D*_1_ into the two partitions $D_{1}^{\prime }$ and $D_{2}^{\prime }$ based on the distribution of queries within *D*_1_. The number of data points in $D_{1}^{\prime }$ and $D_{2}^{\prime }$ is 22 and 28, respectively, and the number of queries are 12 and 18, respectively. Therefore, the new runtime is reduced to ≈200+25 because *D*_1_'s runtime is reduced to ≈100 based on Equation (4). Therefore, the two new data partitions $D_{1}^{\prime }$ and $D_{2}^{\prime }$ are introduced in place of *D*_1_. Next, Partition *D*_2_ is chosen to be split into two partitions, and the optimized runtime becomes ≈100+15. Finally, the procedure terminates as only one available partition is left.

## 4. Local Execution

Once the query plan is generated, each computation node chooses a specific local execution plan based on the queries assigned to it and the indexes it has. We implement various centralized algorithms for spatial range join and *k*NN join operators within each worker and study their performance. The algorithms are implemented in Spark. We use the execution time as the performance measure.

### 4.1. Spatial Range Join

We implement two algorithms for spatial range join (Sowell et al., 2013). The first is indexed nested-loops join, where we probe the spatial index repeatedly for each outer tuple (or range query in the case of shared execution). The tested algorithms are nestRtree, nestGrid, and nestQtree, where they use an R-tree, a Grid, and a Quadtree as an index for the inner table, respectively. The second algorithm is based on the dual-tree traversal (Brinkhoff et al., 1993). It builds two spatial indexes (e.g., an R-tree) over both the input queries and the data, and performs a depth-first search over the dual trees simultaneously.

### 4.2. kNN Join

Similar to the spatial range join, indexed nested-loops can be applied to *k*NN join, where it computes the set of *k*NN objects for each query point in the outer table. An index is built on the inner table (the data table). The other kinds of *k*NN join algorithms are block-based. They partition the queries (the outer table) and the data points (the inner table) into different blocks and find the *k*NN candidates for queries in the same block. Then, post-processing refines step computes *k*NN for each query point in the same block. Gorder (Xia et al., 2004) divides query and data points into different rectangles based on the G-order, and utilizes two distance bounds to reduce the processing of unnecessary blocks. For example, the min-distance bound is the minimum distance between the rectangles of the query points and the data points. The max-distance bound is the maximum distance from the queries to their *k*NN sets. If the max-distance is smaller than the min-distance bound, the related data block is pruned. PGBJ (Lu et al., 2012) has a similar idea that extends to parallel *k*NN join using MapReduce.

Recently, Spitfire (Chatzimilioudis et al., 2016) is a parallel *k*NN self-join algorithm for in-memory data. It replicates the possible *k*NN candidates into its neighboring data blocks. Both PGBJ and Spitfire are designed for parallel *k*NN join, but they are not directly applicable to indexed data. The reason is that PGBJ partitions queries and data points based on the selected pivots while Spitfire is specifically optimized for *k*NN self-join.

LocationSpark enhances the performance of the local *k*NN join procedure. For Gorder (Xia et al., 2004), instead of using the expensive principal component analysis (PCA) in Gorder, we apply the Hilbert curve to partition the query points. We term the modified Gorder method *sfcurve*. We modify PGBJ as follows. First, we compute the pivots of the query points based on a clustering algorithm (e.g., k-means) oversample data, and then partition the query points into different blocks based on the computed pivots. Next, we compute the MBR of each block. Because the data points are already indexed (e.g., using an R-tree), the min-distance from the MBRs of the query points and the index data is computed, and the max-distance bound is calculated based on the *k*NN results from the pivots. This approach is termed *pgbjk*. For Spitfire, we use a spatial index to speed up finding the *k*NN candidates.

## 5. Spatial Bitmap Filter

In this section, we introduce a new spatial bitmap filter termed *sFilter*. The sFilter helps us decide for an outer tuple, say *q*, in a spatial range join, if there exist tuples in the inner table that join with *q*. This helps reduce the communication overhead. For example, consider an outer tuple *q* of a spatial range join where *q* has a range that overlaps multiple data partitions of the inner table. Typically, all the overlapping partitions need to be examined by communicating *q*'s range to them and searching the data within each partition to test for overlap with *q*'s range. This incurs high communication and search costs. Using the sFilter, given *q*'s range that overlaps multiple partitions of the inner table, the sFilter can decide which overlapping partitions contain data that overlaps *q*'s range without actually communicating with and searching the data in the partitions. Only the partitions that contain data that overlap *q*'s range are the ones that will be searched.

**Figure 4:**
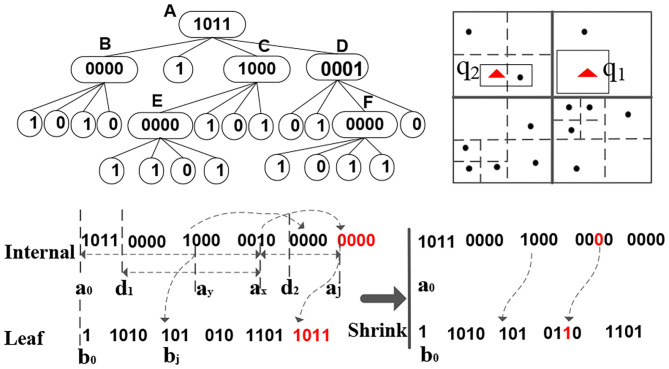
sFilter structure (up left), the related data (up right), and the two bit sequences of the sFilter (down).

Figure 4 gives an example sFilter. Conceptually, an sFilter is a new in-memory variant of a quadtree that has internal and leaf nodes (Samet, 2005). Internal nodes are for index navigation, and leaf nodes, each has a marker to indicate whether or not there are data items in the node's corresponding region. We encode the sFilter into two binary codes and execute queries over this encoding.

### 5.1. Binary Encoding of the sFilter

The sFilter is encoded into two long sequences of bits. The first bit-sequence corresponds to internal nodes while the second bit-sequence corresponds to leaf nodes. Notice that in these two binary sequences, no pointers are needed. Each internal node of the sFilter takes four bits, where each bit represents one of the internal node's children. These children are encoded in clockwise order starting from the upper-left corner. Each bit value of an internal node determines the type of its corresponding child, i.e., whether the child is internal (a 1 bit) or leaf (a 0 bit). In Figure 4, the root (internal) node *A* has binary code 1011, i.e., it has three of its children being internal nodes, and its second node is a leaf node. The four-bit encodings of all the internal nodes are concatenated to form the internal-node bit-sequence of the sFilter. The ordering of the internal nodes in this sequence is based on a breadth-first search (BFS) traversal of the quadtree. In contrast, a leaf node only takes one bit, and its bit value indicates whether or not data points exist inside the spatial quadrant corresponding to the leaf. In Figure 4, Internal Node *B* has four children, and the bit values for *B*'s leaf nodes are 1010, i.e., the first and third leaf nodes of *B* contain data items. During the BFS on the underlying quad-tree of the sFilter, simultaneously construct the bit-sequences for all the leaf and internal nodes. The sFilter is encoded into the two binary sequences in Figure 4. The space usage of an sFilter is ((4^*d*−1^−1)/3) × 4+4^*d*−1^ Bits, where (4^*d*−1^−1)/3 and 4^*d*−1^ are the numbers of internal nodes and leaf nodes, respectively, and *d* = *o*(log(*L*)) is the depth of quadtree and *L* is the length of the space.

### 5.2. Query Processing Using the sFilter

Consider the internal node *D* in Figure 4. *D*'s binary code is 0001, and the fourth bit has Value 1 at Memory Address *a*_*x*_ of the internal-nodes bit-sequence. Thus, this bit refers to *D*'s child *F* that is also an internal node at Address *a*_*j*_. Because the sFilter has no pointers, we need to compute *F*'s address *a*_*j*_ from *a*_*x*_. Observe that the number of bits with Value 1 from Start Address *a*_0_ of the binary code to *a*_*x*_ can be used to compute the address.

*Definition 6* . Let *a* be the bit sequence that starts at Address *a*_0_. χ(*a*_0_, *a*_*x*_) and τ(*a*_0_, *a*_*x*_) are the number of bits with Values 1 and 0, respectively, from Addresses *a*_0_ to *a*_*x*_ inclusive.

χ(*a*_0_, *a*_*x*_) is the number of internal nodes up to *a*_*x*_. Thus, Address *a*_*j*_ of *F* is (*a*_0_+5 × 4) because there are 5 bits with value 1 from *a*_0_ to *a*_*x*_. Similarly, if one child node is a leaf node, its address is inferred from τ(*a*_0_, *a*_*x*_) as follows:

*Proposition 1*. *Let a and b be the sFilter's bit sequences for internal and leaf nodes in Memory Addresses a_0_ and b_0_, respectively. To access a node's child in memory, we need to compute its address. The address, say a_j_, of the xth child of an internal node at Address a_x_ is computed as follows. If the bit value of a_x_ is 1, then a_j_ = a_0_+4 × χ(a_0_, a_x_). If the bit value of a_x_ is 0, a_j_ = b_0_+τ(a_0_, a_x_)*.

We adopt the following two optimizations to speedup the computation of χ(*a*_0_, *a*_*x*_) and τ(*a*_0_, *a*_*x*_): (1) Precomputation and (2) Set counting. Let *d*_*i*_ be the memory address of the first internal node at Height (or Depth) *i* of the underlying quadtree when traversed in BFS order. For example, in Figure 4, Nodes *B* and *E* are the first internal nodes in BFS order at Depths 1 and 2 of the quadtree, respectively. For all *i* ≤ depth of the underlying quadtree, we precompute χ(*a*_0_, *d*_*i*_), e.g., χ(*a*_0_, *d*_1_) and χ(*a*_0_, *d*_2_) in Figure 4. Notice that *d*_0_ = *a*_0_ and χ(*a*_0_, *d*_0_) = 0. Then, Address *a*_*j*_ that corresponds to the memory address of the *x*th child of an internal node at Address *a*_*x*_ can be computed as follows. *a*_*j*_ = *a*_0_+(χ(*a*_0_, *d*_1_)+χ(*d*_1_, *a*_*x*_)) × 4. χ(*a*_0_, *d*_1_) is precomputed. Thus, we only need to compute on the fly χ(*d*_1_, *a*_*x*_). Furthermore, evaluating χ can be optimized by a bit set counting approach, i.e., a lookup table or a sideways addition^1^ that can achieve constant-time complexity.

After getting one node's children via Proposition 1, we apply Depth-First Search (DFS) over the binary codes of the internal nodes to answer a spatial range query. The procedure starts from the first four bits of Bit Sequence *a*, since these four bits are the root node of the sFilter. Then, we check the four quadrants, say *r*_*s*_, of the children of the root node, and iterate over *r*_*s*_ to find the quadrants, say $r_{s}^{\prime }$, overlapping the input query range *q*_*i*_. Next, we continue searching the children of $r_{s}^{\prime }$ based on the addresses computed from Proposition 1. This recursive procedure stops if a leaf node is found with Value 1, or if all internal nodes are visited. For example, consider Range Query *q*_2_ in Figure 4. We start at Root Node *A* (with Bit Value 1011). Query *q*_2_ is located inside the northwestern (NW) quadrant of *A*. Because the related bit value for this quadrant is 1, it indicates an internal node type, and it refers to Child Node *B*. Node *B*'s memory address is computed by *a*_0_+1 × 4 because only one non-leaf node (*A*) is before *B*. *B*'s related bit value is 0000, i.e., *B* contains four leaf nodes. The procedure continues until finding one leaf node of *B*, mainly the southeastern child leaf node, with Value 1 that overlaps the query, and thus returns True.

### 5.3. sFilter in LocationSpark

Since the depth of the sFilter affects query performance, it is impractical to use only one sFilter in a distributed setting. Thus, we embed multiple sFilters into the global and local spatial indexes in LocationSpark. In the master node, separate sFilters are placed into the different branches of the global index, where the role of each sFilter is to locally answer the query for the specific branch it is in. In the local computation nodes, an sFilter is built, and it adapts its structure based on data updates and changes in query patterns.

Algorithm 2 gives the procedure for performing the spatial range join using the sFilter. Initially, the outer (queries) table is partitioned according to the global index. The global index identifies the overlapping data partitions for each query *q*. Then, the sFilter tells which partitions contain data that overlap the query range (Line 2 of the algorithm). After performing the spatial range join (Line 14), the master node fetches the updated sFilter from each data worker, and refreshes the existing sFilters in the master node (Lines 15–16). Lines 2–13 update the sFilter of each worker (as in Figure 2).


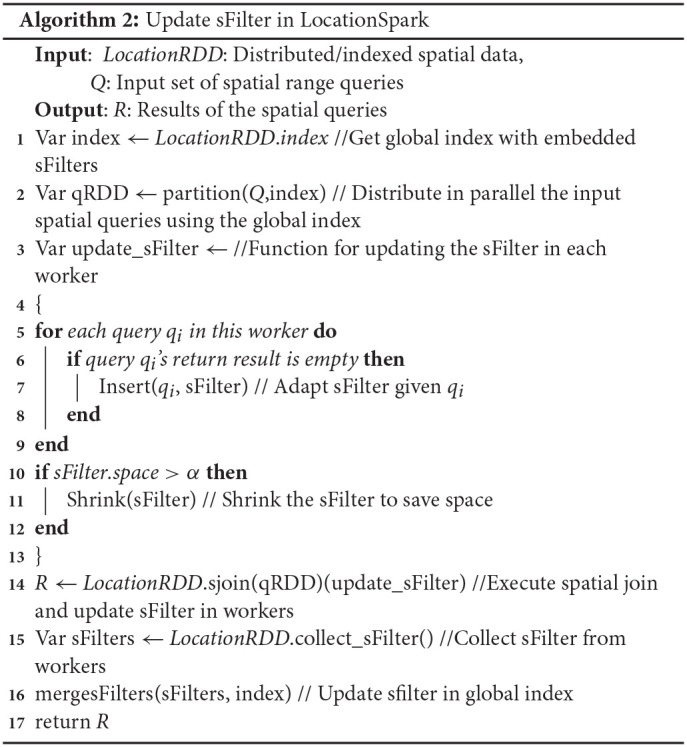


The sFilter can improve the *k*NN search and *k*NN join because they also depend on spatial range search. Moreover, their query results may enhance the sFilter by lowering the false positive errors as illustrated below.

### 5.4. Query-Aware Adaptivity of the sFilter

The build and update operations of the sFilter are first executed at the local workers in parallel. Then, the updated sFilters are propagated to the master node.

The initial sFilter is built from a temporary local quadtree (Samet, 2005) in each partition. Then, the sFilter is adapted based on the query results. For example, consider Query *q*_1_ in Figure 4. Initially, the sFilter reports that there is data for *q*_1_ in the partitions. When *q*_1_ visits the related data partitions, it finds that no data points are overlapping *q*_1_ in the partitions, i.e., a false-positive (+ve) error. Thus, we mark the quadrants precisely covered by *q*_1_ in the sFilter as empty, and hence reduce the false positive errors if queries visit the marked quadrants again. Function insert in Algorithm 2 recursively splits the quadrants covered by the empty query, and marks these generated quadrants as empty. After each local sFilter is updated in each worker, these updates are reflected into the master node. The compact encoding of the sFilter saves the communication cost between the workers and the master.

However, the query performance of the sFilter degrades as the size of the index increases. Function shrink in Algorithm 2 merges some branches of the sFilter at the price of increasing false-positive errors. For example, one can shrink Internal Node *F* in Figure 4 into a leaf node, and updating its bit value to 1, although one quadrant of *F* does not contain data. Therefore, we might track the visit frequencies of the internal nodes, and merge internal nodes with low visiting frequency. Then, some well-known data caching policies, e.g., LRU or MRU, can be used. However, the overhead to track the visit frequencies is expensive. In our implementation, we adopt a simple bottom-up approach. We start merging the nodes from the lower levels of the index to the higher levels until the space constraint is met. In Figure 4, we shrink the sFilter from Internal Node *F*, and replace it by a leaf node, and update its binary code to 1. *F*'s leaf children are removed. The experimental results show that this approach increases the false-positive errors, but enhances the overall query performance.

## 6. Performance Study

LocationSpark is implemented on top of Resilient Distributed Datasets (RDDs); these key components of Spark are fault-tolerant collections of elements that can be operated on in parallel. LocationSpark is a library of Spark, and provides the Class LocationRDD for spatial operations (Mingjie et al., 2016). Statistics are maintained at the driver program of Spark, and the execution plans are generated at the driver. Local spatial indexes are persisted in the RDD data partitions, while the global index is realized by extending the interface of the RDD data partitioner. The data tuples and related spatial indexes are encapsulated into the RDD data partitions. Thus, Spark's fault tolerance naturally applies to in LocationSpark. The spatial indexes are immutable and are implemented based on the path copy approaches. Thus, each updated version of the spatial index can be persisted into a disk for fault tolerance. This enables the recovery of a local index from disk in case of failure in a worker. The Spark cluster is managed by YARN, and a failure in the master nodes is detected and managed by ZooKeeper. In the case of a master node failure, the lost master node is evicted and a standby node is chosen to recover the master. As a result, the global index and the sFilter in the master node are recoverable. Finally, the built spatial index data can be stored into a disk, and enables further data analysis without additional data repartitioning or indexing. LocationSpark is open-source, and can be downloaded from https://github.com/merlintang/SpatialSpark.

### 6.1. Experimental Setup

Experiments are conducted on two datasets. **Twitter**: 1.5 Billion Tweets (around 250 GB) are collected over a period of nearly 20 months (from January 2013 to July 2014), and is restricted to the USA spatial region. The format of a tweet is the identifier, timestamp, longitude-latitude coordinates, and text. **OSMP**: is shared by the authors of SpatialHadoop (Eldawy and Mokbel, 2015). OSMP represents the map features of the whole world, where each spatial object is identified by its coordinates (longitude, latitude) and an object ID. It contains 1.7 Billion points with a total size of 62.3GB. We generate two types of queries. (1) Uniformly distributed (USA, for short): We uniformly sample data points from the corresponding dataset, and generate spatial queries from the samples. These are the default queries in our experiments. (2) Skewed spatial queries: These are synthesized around specific spatial areas, e.g., Chicago, San Francisco, New York (CHI, SF, NY, respectively, for short). The spatial queries and data points are the outer and inner tables *Q* and *D* for the experimental studies of the spatial range and *k*NN joins presented below.

Our study compares LocationSpark with the following: (1) **GeoSpark** (Yu et al., 2015) uses ideas from SpatialHadoop, but is implemented over Spark. (2) **SpatialSpark** (You, 2015) performs partition-based spatial joins. (3) **Magellan** (Hortonworks, 2015) is developed based on Spark's dataframes to take advantage from Spark SQL's plan optimizer. However, Magellan does not have spatial indexing. (4) **State-of-art** **kNN-join**: Since none of the three systems support *k*NN join, we compare LocationSpark with a state-of-art *k*NN-join approach (PGBJ; Lu et al., 2012) that is provided by PGBJ's authors. (5) **Simba** (Xie et al., 2016) is a spatial computation system based on Spark SQL with spatial distance join and *k*NN-join operator. We also modified Simba with the developed techniques (e.g., query scheduler and sFilter) inside, the optimized Simba is called **Simba(opt)**. (6) **LocationSpark(opt)** and **LocationSpark** refers to the query scheduler and sFilter is applied or not, respectively. We do not compare against Stark (Hagedorn et al., 2017), since GeoSpark shows better performance than Stark in most of the cases.

We use a cluster of six physical nodes Hathi^2^. that consists of Dell compute nodes with two 8-core Intel E5-2650v2 CPUs, 32 GB of memory, and 48TB of local storage per node. Meanwhile, in order to test the scalability of LocationSpark, we set up one Hadoop cluster (with Hortonworks data platform 2.5) on the Amazon EC2 with 48 nodes, each node has an Intel Xeon E5-2666 v3 (Haswell) and 8 GB of memory. Spark 1.6.3 is used with YARN cluster resource management. Performance is measured by the average query execution time.

### 6.2. Spatial Range Select and Join

Table 1 summarizes the spatial range select and spatial index build time by the various approaches. For a fair comparison, we cache the indexed data into memory and record the spatial range query processing time. From Table 1, we observe the following: (1) LocationSpark is 50 times better than Magellan on the query execution time for the two tables, mainly because the global and local spatial indexes of LocationSpark can avoid visiting unnecessary data partitions. (2) LocationSpark with different local indexes, e.g., the R-tree and Quadtree, outperforms SpatialSpark. The speedup is around 50 times since SpatialSpark (without index) has to scan all the data partitions. SpatialSpark (with index) stores the global indexes into a disk and finds data partitions by scanning the global index in the disk. This incurs extra I/O overhead. Also, the local index is not utilized during local searching. (3) LocationSpark is around 10 times faster than GeoSpark in spatial range search execution time because GeoSpark does not utilize the built global indexes and scans all data partitions. (4) The local index with Quadtree for LocationSpark achieves superior performance over the R-tree index in terms of index construction and query execution time as discussed in section 4. (5) The index build time among the three systems is comparable because they all scan the data points, which is the dominant factor, and then build the index in memory. (6) LocationSpark achieves 5 times speedup against Simba since sFilter reduces redundant search of data partitions. This is also observed from Simba(opt) (i.e., with sFilter) that can achieve comparable performance with LocationSpark.

**Table 1:** Performance of the spatial range select.

**Dataset**	**System**	**Query time(ms)**	**Index build time(s)**
Twitter	LocationSpark(R-tree)	390	32
	LocationSpark(Qtree)	**301**	16
	Magellan	15,093	/
	SpatialSpark	16,874	35
	SpatialSpark(no-index)	14,741	/
	GeoSpark	4,321	45
	Simba	1,231	34
	Simba (opt)	430	35
OSMP	LocationSpark(R-tree)	1,212	67
	LocationSpark(Qtree)	**734**	18
	Magellan	41,291	/
	SpatialSpark	24,189	64
	SpatialSpark(no-index)	17,210	/
	GeoSpark	4,781	87
	Simba	1,345	68
	Simba(opt)	876	68

*The bold font just emphasizes the system that has the shortest query reponse time*.

**Figure 5:**
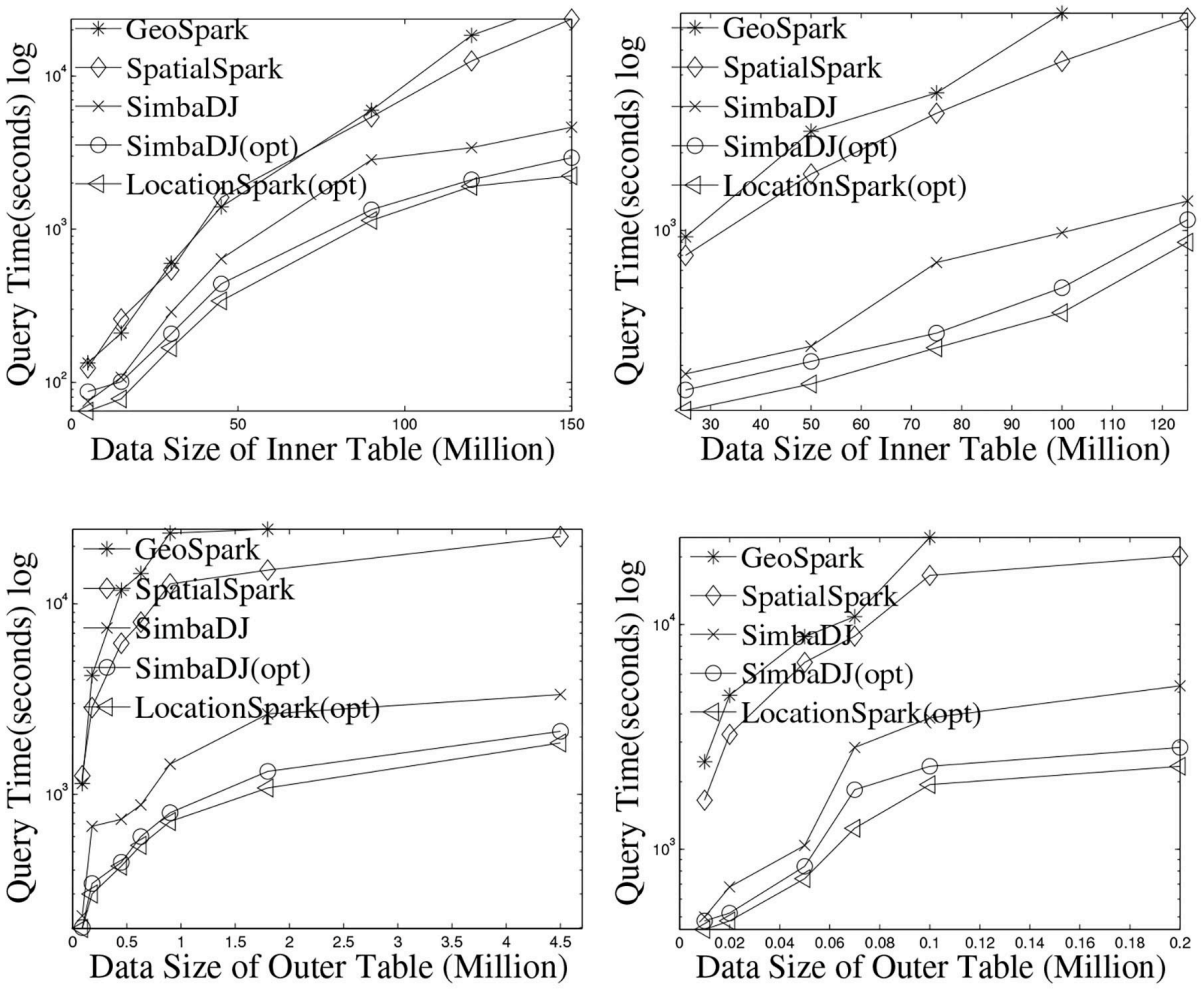
The performance of spatial range join on dataset: Twitter and OSMP.

The execution times of the spatial range join are listed in Figure 5. For a fair comparison, the runtime includes the time to initiate the job, build indexes, execute the join query, and save results into HDFS. Note that Simba does not support spatial range join, it only provides the spatial distance join, where each query is a rectangle or circle with the same size. Performance results for Magellan are not shown because it performs a Cartesian product and hence has the worst execution time. Figure 5 presents the results by varying the data sizes of *D* (the inner table) from 25 to 150 million, while keeping the size of *Q* (the outer table) to 0.5 million. The execution time of GeoSpark shows a quadratic increase as the data size increases. GeoSpark's running time is almost 3 h when the data size is 150 million, which is extremely slow. SpatialSpark shows similar trends. The reason is that both GeoSpark and SpatialSpark suffer from the following: (1) The spatial skew issue, where some workers process more data and take a longer time to finish. (2) The local execution plans based on the R-tree and the Grid are slow. (3) Query processing accesses data partitions that do not contribute to the final results. LocationSpark with the optimized query plans and the sFilter outperforms the two other systems by an order of magnitude. Also, we study the effect of the outer table size on performance. Figure 5 give the run time, and demonstrate that LocationSpark is 10 times faster than the other two systems. From Figure 5, we also observe that Simba outperforms GeoSpark and SpatialSpark more than one order of magnitude. However, Simba suffers from query skew issues and degrades the performance quickly, because Simba duplicates each data point into multiple data partitions if it spatially overlaps the query rectangles. Naturally, the bigger the spatial query rectangle, the more data points to be duplicated. This introduces redundant network and computation costs. Thus, we place the query scheduler and sFilter into the physical execution part of Simba (e.g., modifying the RDKspark). From the experimental results, the performance of Simba is improved dramatically. This also proves that the proposed approaches in this work could be used to improve other in-memory distributed spatial query processing systems.

### 6.3. Performance of kNN Select and Join

**Figure 6:**
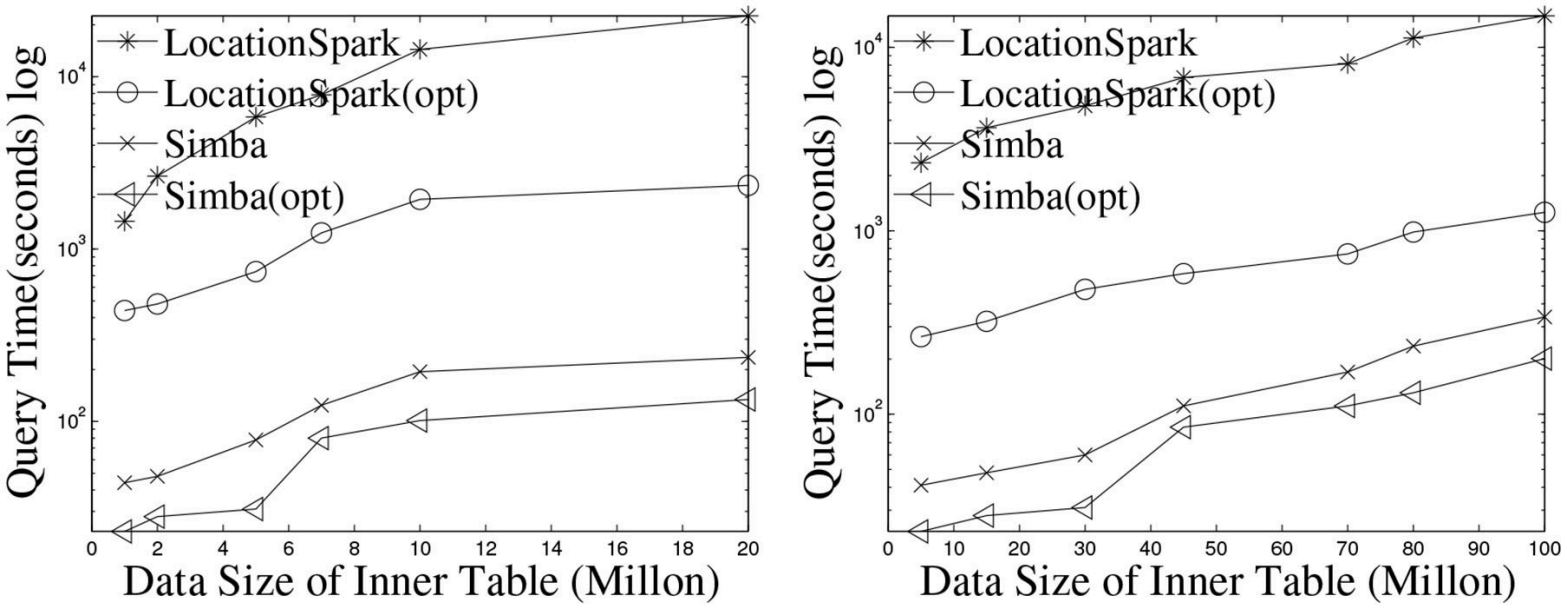
Performance of *k*NN join by increasing the number of data points for OSMP **(left)** and Twitter **(right)**.

Performance of *k*NN select is given in Figure 6. LocationSpark outperforms GeoSpark by an order of magnitude. GeoSpark broadcasts the query points to each data partition and accesses each data partition to get the *k*NN set for the query. Then, GeoSpark collects the local results from each partition, then sorts the tuples based on the distance to a query point of *k*NN. This is prohibitively expensive and results in large execution times. LocationSpark only searches for data partitions that contribute to the *k*NN query point based on the global and local spatial indexes and the sFilter. It avoids redundant computations and unnecessary network communication for irrelevant data partitions.

**Figure 7:**
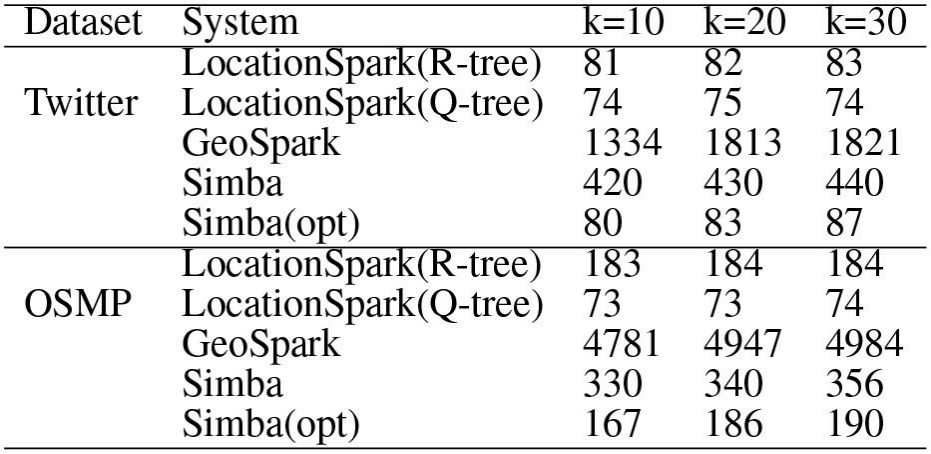
*k*NN select (in ms).

For *k*NN join, Figure 7 gives the performance results when varying *k* on Twitter and OSMP datasets. In terms of runtime, LocationSpark with optimized query plans and with the sFilter always perform the best. LocationSpark without any optimizations gives better performance than that of PGBJ. The reason is due to having in-memory computations and avoiding expensive disk I/O when compared to MapReduce jobs. Furthermore, LocationSpark with optimization shows around 10 times speedup over PGBJ, because the optimized plan migrates and splits the skewed query regions.

**Figure 8:**
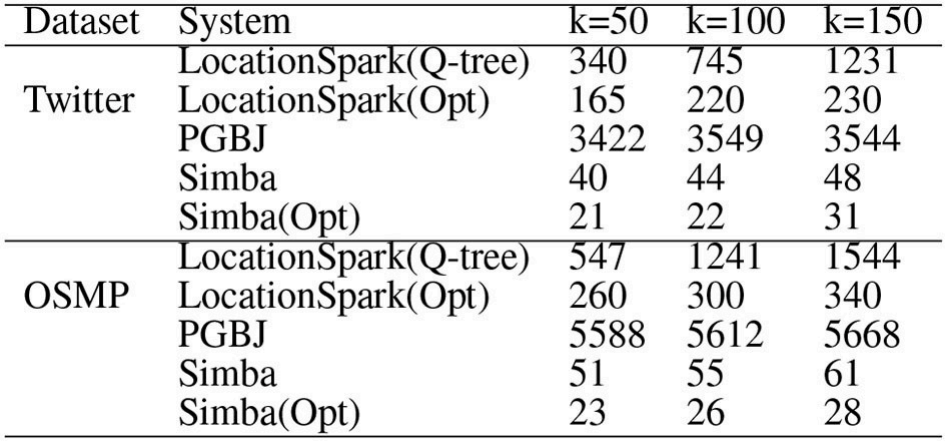
Runtime of *k*NN join (in seconds).

We test the performance of the *k*NN join operator when increasing the number of data points while having the number of queries fixed to 1 million around the Chicago area. The results are illustrated in Figure 8. Observe that LocationSpark with optimizations performs an order of magnitude better than the basic approach. The reason is that the optimized query plan identifies and repartitions the skewed partitions. In this experiment, the top five slowest tasks in LocationSpark without optimization take around 33 min, while more than 75% tasks only take <30 s. On the other hand, with an optimized query plan, the top five slowest tasks take <4 min. This directly enhances the execution time.

Observe that Simba outperforms LocationSpark around 10 times for the *k*NN join operation. This speedup is achieved by implementing the *k*NN join via a sampling technique in Simba. More specifically, Simba at first samples query points, and computes the *k*NN join bound to prune data partitions that do not contribute *k*NN join results. The computed *k*NN bound could be very tight when *k* is small and the query points are well-balanced and distributed. More details can be found in Simba (Xie et al., 2016).

However, the computed *k*NN join bound has to check for spatial overlap with data points, then duplicate the query points to the overlapping data partitions as well. As a result, Simba would suffer from query skew, where certain data partitions are overwhelmed with query points. Furthermore, a bigger *k* value introduces redundant network communication because the *k*NN join bound would be bigger. Similar to spatial join, we place the spatial query scheduler and the sFilter inside the physical execution part of Simba. The query scheduler can detect the skewed data partitions, and the sFilter removes the data partitions that overlap the *k*NN join bound but has no contributing results. Therefore, the performance of Simba is further improved, e.g., more than three times in this experimental setting.

Furthermore, we study the performance under various query distributions, the number of execution nodes, and the effect of the sFilter. More details are presented in the technical report (Mingjie et al., 2016). In addition, most recent work (Pandey et al., 2018) compare the performance of big spatial data processing system, and it proves LocationSpark shows much better performance in different aspects.

## 7. Related Work

Spatial data management has been extensively studied for decades and several surveys provide good overviews. Gaede and Günther (1998) provides a good summary of spatial data indexing. Sowell et al. (2013) present a survey and experimental study of iterative spatial-join in memory. Recently, there has been considerable interest in supporting spatial data management over Hadoop MapReduce. Hadoop-GIS (Aji et al., 2013) supports spatial queries in Hadoop by using a uniform grid index. SpatialHadoop (Eldawy and Mokbel, 2015) builds global and local spatial indexes, and modifies the HDFS record reader to read data more efficiently. MD-Hbase (Nishimura et al., 2011) extends HBase to support spatial data update and queries. Hadoop MapReduce is good at data processing for high throughput and fault-tolerance.

Taking advantage of the very large memory pools available in modern machines, Spark and Spark-related systems (e.g., Graphx, Spark-SQL, and DStream) (Gonzalez et al., 2014; Zaharia, 2016) are developed to overcome the drawbacks of MapReduce in specific application domains. To process big spatial data more efficiently, it is natural to develop efficient spatial data management systems based on Spark. Several prototypes have been proposed to support spatial operations over Spark, e.g., GeoSpark (Yu et al., 2015), SpatialSpark (You, 2015), Magellan (Hortonworks, 2015), Simba (Xie et al., 2016), Stark (Hagedorn et al., 2017). However, some important factors impede the performance of these systems, mainly, query skew, lack of adaptivity, and excessive and unoptimized network and I/O communication overheads. For existing spatial join (Brinkhoff et al., 1993; Sowell et al., 2013) and *k*NN join approaches (Xia et al., 2004; Lu et al., 2012; Chatzimilioudis et al., 2016), we conduct experiments to study their performance in section 4. The reader is referred to section 4. For testing how the proposed techniques to improve Simba, we placed the spatial query scheduler and the sFilter inside Simba standalone version. Simba's standalone version is based on Spark SQL Dataframe while removing the support of the Spark SQL parser. The query scheduler and the sFilter are placed inside the physical plan of Simba (i.e., RDJSpark and RKJSpark). For spatial data updating, it belongs to data processing engine scope but related to data storage like (Memarzia et al., 2019). To support querying over polygonal or polyline objects, we can reuse the spatial index of LocationSpark (e.g., the local and global indexes), then extend the workload scheduling model and sFilter while considering the properties of polylines. More specifically, a polygon is typically represented by a rectangle that is enclosing the polgyon. The rectangles are used for “filtering.” Anything that is outside the rectangle is guaranteed not to intersect the polygon, Once a query intersects the rectangle, we need to apply a detailed polygon intersection, which is relatively expensive. We can use the sFilter to indicate the empty space between the polygon and its representing rectangle, and this would reduce the computation cost.

Kwon et al. (2010, 2012) proposes a skew handler to address the computation skew in a MapReduce platform. AQWA (Aly et al., 2015) is a disk-based approach that handles spatial computation skew in MapReduce. In LocationSpark, we overcome the spatial query skew for spatial range join and *k*NN join operators and provide an optimized query execution plan. These operators are not addressed in AQWA. The query planner in LocationSpark is different from relational query planners, i.e., join order and selection estimation. ARF (Alexiou et al., 2013) supports a one-dimensional range query filter for data in the disk. Calderoni et al. (2015) study spatial Bloom filter for private data. Yet, it does not support spatial range querying.

## 8. Conclusions

We present LocationSpark, a query executor, and an optimizer based on Spark to improve the query execution plan generated for spatial queries. We introduce a new spatial bitmap filter to reduce the redundant network communication cost. Empirical studies on various real datasets demonstrate the superiority of our approaches compared with existing systems. In the future, we would like to adopt the introduced techniques of LocationSpark into the Spark SQL optimization engine, and investigate how spatial operations enabled with the spatial bitmap filter would work with relational data operators.

## Data Availability Statement

The datasets generated for this study are available on request to the corresponding author.

## Author Contributions

MT and YY contributed to the design, implementation, and analysis of the results in the manuscript. AM, QM, MO, and WA contributed to the writing of the manuscript. WA also contributed to the design and analysis of the manuscript, in addition to the contribution of writing the manuscript. All authors contributed to the article and approved the submitted version.

## Conflict of Interest

YY was employed by the company Facebook. AM was employed by the company Google. The remaining authors declare that the research was conducted in the absence of any commercial or financial relationships that could be construed as a potential conflict of interest.

## Footnotes

^1^https://graphics.stanford.edu/~seander/bithacks.html

^2^https://www.rcac.purdue.edu/compute/hathi/
